# Supplementary material for: A generalized theory of age-dependent carcinogenesis
Source: eLife. 2019 Apr 29;8:e39950. doi: 10.7554/eLife.39950 (PMC6488293; doi:10.7554/eLife.39950)
Supplement: Supplementary file 1. [file elife-39950-supp1.docx]

**Model Matlab code** *(use Figure 2A for a visual guide)*

growthCoeff = 0.007; % coefficient of HSC pool growth corresponding to body growth

tier3 = 1182046340; % the size of the Tier 3 genome

DnaMutRate = 10^-8; % rate of mutation per cell division

%VARIABLE RANGES (contain tested ranges of factors determining somatic evolution)

nDriversRange = [1 2 3 4]; % minimum number of drivers required

mutFitCurveRange = [0.001 0.0025 0.005 0.01];

% constant fitness advantage conferred by driver mutations (MMC model)

envDeclRange = [10 30 50 70];

% coefficient of aging curve dynamics (MMC-DS model) influencing

% age-dependent changes in selection as sown in Fig 2C (lower chart)

strNegSelRange = [-0.5 -0.3 -0.1 0];

% coefficient of negative to positive selection ratio (MMC-DS model)

% determining the relative strength of early life negative selection and

% late-life positive selection as shown in Fig 2C (middle chart)

strGenSelRange = [0.001 0.005 0.01 0.05];

% coefficient of the general selection strength (MMC-DS model) determing

% the strength of both early life negative and late-life positive selection

% as shown in Fig 2C (upper chart)

adultCycleRange = [40 50 60 70];

% adult cell division rate (~weeks between successive cell divisions)

maxHSCRange = [10000 25000 50000 100000];

% adult simulated cell pool size in number of cells

phenMutRateRange = [10^-2 10^-3 10^-4 10^-5];

% effective phenotypic mutation rate (rate of driver mutations per division)

lifespanRange = [100*52];

% total simulattion time in weeks

%>>>>>>>>>>>>>>>>>>>

nConds = 4; % 4 simulated conditions according to four values of each parameter range described above

%>>>>>>>>>>>>>>>>>>>

% outermost loop runs simulations for each of the four values of the tested

% parameter of the parameter ranges described above

for c = 1 : nConds

%LOCAL PARAMETERS

runs = 50; % 50 repeats of each simulation condition to collect statistics

initHSC = round(300/maxHSCRange(1)*maxHSCRange(c)); % initial postnatal number of simulated HSCs in the HSC pool

initCycle = 3;

%========================================

% each parameter in the simulation is set to its default value of the 4

% possible values within the parameter's range (described above). One

% parameter is tested with the "c" iterator to explore the parameter's

% influence on the model within its range (4 values)

maxHSC = maxHSCRange(1); % adult HSC pool capacity in number of cells

maxAge = lifespanRange(end); % longevity of the simulated individual

adultCycle = adultCycleRange(1); % adult HSC cell division rate (weeks between successive divisions)

phenMutRate = phenMutRateRange(c); % rate of driver mutations per cell division

envDecl = envDeclRange(1); % the curve of aging influencing the dynamics of age-dependent selection shift in the MMC-DS model

strNegSel = strNegSelRange(2); % strength of early life negative selection relative to late-life positive selection as shown in Fig 2C (middle chart)

strGenSel = strGenSelRange(2); %general strength of selection as shown in Fig 2C (upper chart)

fitnValStable = true; % boolean determining if the simulated run operates with fixed value of driver mutation fitness effects (MMC model) or changing age-dependent effect (MMC-DS model)

stabFitVal = mutFitCurveRange(1); % currently simulated fitness value of a driver mutation if fixed stable value (MMC model) is assumed

%=====================================

%stable or dynamic fitness value of oncogenic mutations

if(fitnValStable)

mutFitCurve(1, 1 : maxAge) = stabFitVal; % if fixed fitness value of a driver mutation is assumed, it remains the same throughout life

else

mutFitCurve = ((1 ./ (1+exp(-0.0028.*...

([1:maxAge]-(envDecl*52))))) + strNegSel).*strGenSel; % if age-dependent fitness value of driver mutations is assumed, it changes proportionally to physiological aging (aging curve)

end

%=================================================================================

% the code block generates the curve of the HSC pool

% capacity in numbers of cells as a function of age (early life growth followed by adult life stable size as shown in Fig 2B upper chart)

oldCapacity = initHSC;

growthCurve = [initHSC];

for i = 1 : maxAge

newCapacity = oldCapacity + (round(growthCoeff...

* oldCapacity * ((maxHSC - oldCapacity) / maxHSC)));

growthCurve = [growthCurve, newCapacity];

oldCapacity = newCapacity;

end

%================================================================================

clearvars HSCpool % cleanup from previous run

for run = 1 : runs

%====================================

% a new postnatal HSC pool is generated containing the initial

% number of cells each with the 4 parameters described below

HSCpool(1, 1 : initHSC) = 0;

HSCpool(2, 1 : initHSC) = randi(initCycle, [1 initHSC]);

HSCpool(3, 1 : initHSC) = 0;

HSCpool(4, 1 : initHSC) = 1;

% 1. ID: wt - 0, driver muts > 0; zero means wild-type cell; each

% driver mutation increments the number by one showing the number

% of mutations driver mutations a cell has acquired

% 2. cell age as the number of weeks after the last division for

% determining when the cell will divide next time

% 3. tier 3 mutations - number of mutations in Tier 3 (neutral mutations)

% 4. cell fitness - initially equal to 1 and subsequently changed

% by driver mutations

%==========================================

% inner loop simulating the life of one individual HSC pool within

% the simulated longevity

for currentAge = 1 : maxAge

currentAge

% current cell division rate depending on age

%as shown in Fig 2B lower chart

cellDiv = initCycle + ((adultCycle-initCycle)...

*(abs(size(HSCpool, 2)-initHSC)/abs(maxHSC-initHSC)));

% CELL DIVISIONS (Step 1 in Figure 2A) - a draw from a normal distribution to

% determine at what time after last division each cell will

% divide based on the current age-dependent average cell

% division rate

divMatrix = normrnd(cellDiv, cellDiv/8, 1, size(HSCpool, 2));

% resets the time past division for cells that divide

% cells that are older than specified in the divMatrix above

% divide

HSCpool(2, HSCpool(2, :) >= divMatrix(1, :)) = 1;

newCells = HSCpool(:, HSCpool(2, :) == 1); %new cells

numCellDivisions(run, currentAge) = size(newCells, 2); % record of the number of cells that divided

% new cells are created originating from that divided

newCells = [newCells, newCells];

HSCpool(:, HSCpool(2, :) == 1) = 0;

HSCpool = HSCpool(:, HSCpool(4, :) > 0);

% TIER 3 (SILENT) MUTATIONS OCCUR (Step 2 in Figure 2A)

% tier 3 mutations occur as a result of cell division with

% corresponding probabilities based on DNA mutation rate

newCells(3, :) = newCells(3, :) + (DnaMutRate*tier3);

% DRIVER MUTATIONS OCCUR (Step 3 in Figure 2A)

% driver mutations occur as a result of cell division with

% corresponding probabilities based on phenotypic mutation rate

newCells(1, :) = newCells(1, :)...

+ binornd(1, phenMutRate, 1, size(newCells, 2));

% CELL FITNESS UPDATED (Step 4 in Figure 2A)

% fitness change as a result of driver mutations

% fitness =

% (initial fitness + fitness effect of one mutation)^(number of

% mutations)

newCells(4, :) =...

(1 + mutFitCurve(1, currentAge)).^newCells(1, :);

% after mutation and fitness change cells are added back to the

% pool

HSCpool = [HSCpool, newCells];

%variable cleanup

newCells = [];

numOncMut = [];

%===========================================================

% CELLS COMPETE FOR THE NICHE (Step 5 in Figure 2A)

% after cell division the pool is larger than allowed by the

% current age-dependent pool size. Cells compete for niche with

% those of higher fitness having higher chances to remain in

% the pool, and those of lower fitness disappear from the pool

% with higher probability (this simulated the combined effect of cell death, senescence and differentiation into the soma)

w_prob = find(HSCpool(2, :));

n = size(w_prob, 2);

w_prob(2, :) = (growthCurve(currentAge) / n)...

* (n * HSCpool(4, w_prob(1, :)) / sum(HSCpool(4, :)));

w_prob(2, w_prob(2,:) >= 1) = 1;

w_prob(3, :) = binornd(1, w_prob(2, :), 1, size(w_prob, 2));

HSCpool(:, w_prob(1, w_prob(3, :) == 0)) = 0;

w_prob = [];

HSCpool = HSCpool(:, HSCpool(4, :) > 0);

%===============================================================

% CELL AGE (time past division) is incremented by one week

HSCpool(2, :) = HSCpool(2, :) + 1;

end % end of individual life simulation

HSCpool = []; % the simulated HSC pool is deleted

end % end of one of 50 repeated runs with the same parameters (statistics collection)

end % end of entire simulation

**Standard parameters sets**

*Standard parameter set MMC:*

Initial cell number: 300

Adult pool size: 10,000

Initial cell division rate: 1 division in ~3 weeks

Adult cell division rate: 1 division in ~40 weeks

Phenotypic mutation rate: 10^-3^

Fixed fitness value of driver mutations: +0.1%

*Standard parameter set MMC-DS:*

Initial cell number: 300

Adult pool size: 10,000

Initial cell division rate: 1 division in ~3 weeks

Adult cell division rate: 1 division in ~40 weeks

Phenotypic mutation rate: 10^-3^

General strength of selection: +/-Fmax = +/-0.5% (**Figure 1C**)

Ratio of early negative to late positive selection: -Fmax/+Fmax = -0.4%/+0.6% (**Figure 1C**)

Aging curve (age of selection sign switch point): 50 years (**Figure 1C**)
