# Supplementary material for: A generalized theory of age-dependent carcinogenesis
Source: eLife. 2019 Apr 29;8:e39950. doi: 10.7554/eLife.39950 (PMC6488293; doi:10.7554/eLife.39950)
Supplement: Transparent reporting form [file elife-39950-transrepform.pdf]

eLIFE

1st Floor
24 Hills Road
Cambridge CB2 1JP, UK

P 01223 855340
W elifesciences.org
T @elife

# *eLife's* transparent reporting form

We encourage authors to provide detailed information *within their submission* to facilitate the interpretation and replication of experiments. Authors can upload supporting documentation to indicate the use of appropriate reporting guidelines for health-related research (see EQUATOR Network), life science research (see the BioSharing Information Resource), or the ARRIVE guidelines for reporting work involving animal research. Where applicable, authors should refer to any relevant reporting standards documents in this form.

If you have any questions, please consult our Journal Policies and/or contact us: editorial@elifesciences.org.

## Sample-size estimation

- You should state whether an appropriate sample size was computed when the study was being designed
- You should state the statistical method of sample size computation and any required assumptions
- If no explicit power analysis was used, you should describe how you decided what sample (replicate) size (number) to use

Please outline where this information can be found within the submission (e.g., sections or figure legends), or explain why this information doesn't apply to your submission:

Our sample size for statistical analyses was 50 repeated simulation runs for most experiments, except those shown in Fig. 5 where the sample size was 25,000 individual runs. Traditional power analysis was not conducted, since our statistics (as shown in supplemental figures for p-values) were done at each point along the 5200 simulation time points (X-axis in all results charts). This approach vastly increases the effective sample size. More importantly, however, variance among the 50 repeated runs varies significantly along the simulated 5200 time points. This would call for various sample sizes (number of runs) depending on a particular simulation setup/experiment and particular time point of each simulation run, which overall makes power analysis pointless. For this reason, we chose arbitrarily to make 50 repeated runs, which is a reasonably large sample (given that statistics were calculated for each of the 5200 time points for each 50 run sample), but small enough to save computational power. For the experiments shown in Fig. 5, we were interested in a sample size large enough in order to be able to catch early life 4-mutation clones shown in Fig. 5B, which are rare events in the model setup (mutation rate, cell division rate and number of cells) and in real life (the frequency of multi-mutation childhood leukemia). Variance of later life events did not matter in these experiments, as it was not subject of investigation in this case

## Replicates

- You should report how often each experiment was performed
- You should include a definition of biological versus technical replication
- The data obtained should be provided and sufficient information should be provided to indicate the number of independent biological and/or technical replicates
- If you encountered any outliers, you should describe how these were handled
- Criteria for exclusion/inclusion of data should be clearly stated
- High-throughput sequence data should be uploaded before submission, with a private link for reviewers provided (these are available from both GEO and ArrayExpress)

Please outline where this information can be found within the submission (e.g., sections or figure legends), or explain why this information doesn't apply to your submission:

In our study, the number of replicates was 50 and 25,000 as described in the explanation for sample size and power analysis. These repeated runs can be considered as an analog of biological replicates, as Monte Carlo simulations inherently involve variance generated by random number distributions, which simulates natural variance. In essence, each simulated run corresponds to one lifespan of one individual

eLife Sciences Publications, Ltd is a limited liability non-profit non-stock corporation incorporated in the State of Delaware, USA, with company number 5030732, and is registered in the UK with company number FC030576 and branch number BR015634 at the address 1st Floor, 24 Hills Road, Cambridge CB2 1JP | August 2014

## Statistical reporting

- Statistical analysis methods should be described and justified
- Raw data should be presented in figures whenever informative to do so (typically when N per group is less than 10)
- For each experiment, you should identify the statistical tests used, exact values of N, definitions of center, methods of multiple test correction, and dispersion and precision measures (e.g., mean, median, SD, SEM, confidence intervals; and, for the major substantive results, a measure of effect size (e.g., Pearson's r, Cohen's d)
- Report exact p-values wherever possible alongside the summary statistics and 95% confidence intervals. These should be reported for all key questions and not only when the p-value is less than 0.05.

Please outline where this information can be found within the submission (e.g., sections or figure legends), or explain why this information doesn't apply to your submission:

In each experiment, we generated a p-value calculated based on the distribution of clonal sizes of the 50 repeated runs at each time point of the simulation (5200 total). These p-values are presented as charts of p-values along the X-axis (time) of the simulation and demonstrate differences in clonal behavior all along the simulation run, i.e. they reflect temporal statistics. Each experiment included 4 different conditions (parameter sets), each 50 repeated runs. All statistical comparisons between these 4 conditions were made using the Kruskal-Wallis method, which is a non-parametric version of ANOVA. We chose non-parametric statics, again, for the reason that we made longitudinal statistical measures, whereby it is difficult to expect normal distribution of individual clonal size along the entire lifespan (X-axis). For uniformity and practicality of analyses, we chose the Kruskal-Wallis method as a safe assumption for analyses where estimation of normality of distribution is impractical. For statistical conclusions, we used the 0.05 p-value cut-off, which is shown in supplemental figures S2-S7. However, as we demonstrate temporal changes in p-values along the entire simulation time, statistical conclusions are relevant only to each given time point (i.e. differences and similarities in clonal abundances change over time).

(For large datasets, or papers with a very large number of statistical tests, you may upload a single table file with tests, Ns, etc., with reference to sections in the manuscript.)

## Group allocation

- Indicate how samples were allocated into experimental groups (in the case of clinical studies, please specify allocation to treatment method); if randomization was used, please also state if restricted randomization was applied
- Indicate if masking was used during group allocation, data collection and/or data analysis

Please outline where this information can be found within the submission (e.g., sections or figure legends), or explain why this information doesn't apply to your submission:

Each tested parameter range was represented by 4 parameter values shown in the code and elsewhere. For instance, adult cell division rates were once in 40, 50, 60, and 70 weeks. Clonal dynamics were then compared among these 4 experimental conditions separately for clones harboring 1, 2, 3, and 4 driver mutations

## Additional data files ("source data")

- We encourage you to upload relevant additional data files, such as numerical data that are represented as a graph in a figure, or as a summary table
- Where provided, these should be in the most useful format, and they can be uploaded as "Source data" files linked to a main figure or table
- Include model definition files including the full list of parameters used
- Include code used for data analysis (e.g., R, MatLab)
- Avoid stating that data files are "available upon request"

Please indicate the figures or tables for which source data files have been provided:

p-values were calculated using the following Matlab code:

```
matrix1 = % data for 1-mutation clones;
matrix2 = % data for 2-mutation clones;
matrix3 = % data for 3-mutation clones;
matrix4 = % data for 4-mutation clones;
runs = 50;
line = 2;
matrix =% matrix1, 2, 3 or 4 (the dataset currently analyzed)
input = [];
inp = zeros(runs, 4);
```
